# Systemic inflammatory markers and neurovascular changes in the retina and choroid of diabetic patients without retinopathy: insights from wide-field SS-OCTA

**DOI:** 10.3389/fmed.2025.1566047

**Published:** 2025-06-04

**Authors:** Qinxing Wu, Bin Zhao, Shengliang Dongye, Lu Sun, Bo An, Qian Xu

**Affiliations:** ^1^Department of Ophthalmology, The Second Affiliated Hospital of Shandong First Medical University, Tai’an, China; ^2^Shandong Healthcare Industry Development Group, Tai’an, China

**Keywords:** systemic inflammation, diabetic retinopathy, early biomarkers, neurovascular changes, wide-field SS-OCTA

## Abstract

**Purpose:**

The purpose of this study was to investigate the association between systemic inflammation markers and early neuronal and microvascular changes in the retinal and choroidal regions of patients with type 2 diabetes mellitus (T2DM) without clinical signs of diabetic retinopathy (DR), utilizing wide-field swept-source optical coherence tomography angiography (SS-OCTA).

**Methods:**

This retrospective, observational cohort study included 61 patients (119 eyes) with T2DM without clinical DR (NDR group) and 44 healthy individuals (82 eyes) as controls. All participants underwent a comprehensive ophthalmic evaluation and blood sampling for hematologic indices. Inflammatory markers, including neutrophil-to-lymphocyte ratio (NLR), platelet-to-lymphocyte ratio (PLR), monocyte-to-lymphocyte ratio (MLR), systemic immune-inflammation index (SII), and systemic inflammation response index (SIRI), were calculated. The mean thickness of the retina, choroid, and individual inner retinal layers, as well as the vessel density measurements of the superficial and deep retinal layers, and the choriocapillaris perfusion area, were recorded and analyzed from the OCTA images. Additionally, the choroidal vascularity index (CVI) was determined.

**Results:**

The NDR group demonstrated significantly higher levels of NLR, SII, and SIRI compared to the control group (*p* < 0.05). The diabetic cohort showed reduced vessel density in the deep capillary plexus (DCP) across all measured regions (*p* < 0.05). Significant but weak negative correlations were observed between inflammation markers, particularly NLR, and OCTA parameters, with a marked impact on the DCP (*r* = −0.21 to −0.32, *p* < 0.05) and CVI (*r* = −0.23 to −0.28, *p* < 0.05).

**Conclusion:**

The study provides new insights into the role of systemic inflammation in early structural and blood flow changes in the retina and choroid, occurring prior to the onset of DR. The findings highlight the importance of inflammation in the pathogenesis of DR, even in the absence of clinical signs, suggesting that systemic inflammatory markers may serve not only as early biomarkers of ocular changes in T2DM but also as potential early therapeutic targets to prevent or delay diabetic retinopathy.

## Introduction

Diabetic retinopathy (DR) is considered one of the leading causes of vision impairment among working-age individuals, and its prevalence is increasing significantly due to the rising rates of diabetes (1, 2). Moreover, epidemiological studies have linked DR not only to visual impairment but to elevated all-cause mortality in diabetic patients (3, 4). Despite extensive research on DR, several challenges remain in fully understanding its pathology and related complications. More recent studies have moved from focusing solely on the microvasculature to exploring the changes in the interdependence within the neurovascular unit (NVU) (5, 6). Achieving a clearer understanding of the temporal and topographical distribution of retinal neurovascular impairment could enable a more comprehensive analysis of disease progression and potentially reveal new therapeutic targets.

Numerous studies involving diabetic patients and animal models have demonstrated that chronic tissue inflammation plays a pivotal role in the pathophysiology of DR (7–9). Sustained hyperglycemia induces localized upregulation of inflammatory molecules, which activate and disrupt the function of glial cells, potentially contributing to early neuroretinal damage in DR (10). Additionally, a recent review has emphasized the value of anti-inflammatory interventions in diabetic ocular complications, underscoring the impact of inflammation on diabetic eye disease (11).

Composite inflammation markers, such as the neutrophil-to-lymphocyte ratio (NLR), platelet-to-lymphocyte ratio (PLR), monocyte-to-lymphocyte ratio (MLR), systemic immune-inflammation index (SII), and systemic inflammation response index (SIRI), are novel biomarkers derived from traditional peripheral blood cell counts. These indices reflect the balance between inflammation and immune response and can be easily and cost-effectively calculated (12, 13). Previous studies have indicated that these systemic inflammation indices are strongly associated with the onset and progression of DR and diabetic macular edema (14–16). However, the relationship between systemic inflammation indices and NVU damage in DR remains to be further explored.

Optical coherence tomography angiography (OCTA) provides high-resolution images of the retinal and choroidal capillary networks, making it essential to diagnose and monitor several ocular diseases. It has become a critical tool in the management of DR (17). Furthermore, the clinical application of wide-field swept-source optical coherence tomography angiography (SS-OCTA) has greatly advanced our understanding of chorioretinal vasculature (18). Particularly in DR, pathological changes beyond the macula are often more critical for a comprehensive disease evaluation.

Building on this background, our study used wide-field SS-OCTA to assess the thickness of retinal neurovascular layers, retinal blood flow density, choroidal thickness, and choroidal vascular index in patients with type 2 diabetes mellitus (T2DM) without clinical DR, compared to healthy controls. Additionally, we explored the relationship between NVU abnormalities and systemic inflammation markers in individuals with preclinical DR.

## Method

### Study population

This retrospective, observational cohort study included adult patients aged 40 to 85 years with T2DM who underwent regular DR screenings between January 2024 and December 2024 at the Department of Ophthalmology, Second Affiliated Hospital of Shandong First Medical University. The control group consisted of healthy subjects without diabetes who visited the clinic for a general health checkup. The study was approved by the Ethics Committee of the Second Affiliated Hospital of Shandong First Medical University (Approval No. 2024-H-049) and conducted in accordance with the principles of the Declaration of Helsinki. Written informed consent was obtained from each subject.

Three trained ophthalmologists (QX, QW, and LS) performed a comprehensive ophthalmologic examination following a standardized protocol, which included obtaining a history of previous ocular diseases, trauma, or surgery; visual acuity; refraction; slit-lamp microscopy; direct ophthalmoscopy; intraocular pressure (IOP) measurement; and fundus photography. Patients with hypertension, hypercholesterolemia, cerebrovascular disease, or cardiovascular disease, as diagnosed by internal medicine physicians, were excluded. Additional exclusion criteria comprised refractive errors exceeding ±3.0 diopters, presence of macular edema or other fundus abnormalities associated with diabetic retinopathy, a history of previous intraocular surgery or laser photocoagulation, treatment with anti-vascular endothelial growth factor (VEGF) agents, or any evidence of other chorioretinal diseases in the studied eye. A total of 61 patients (119 eyes) with T2DM, classified as non-diabetic retinopathy (NDR) (i.e., diabetic patients without clinically significant signs of DR), were recruited. In addition, 44 healthy individuals (82 eyes) with no history of diabetes or ocular disease were recruited as controls.

Three trained ophthalmologists (QX, QW, and LS) performed a comprehensive ophthalmologic examination following a standardized protocol, which included obtaining a history of previous ocular diseases, trauma, or surgery; measurement of visual acuity and refraction; slit-lamp microscopy; direct ophthalmoscopy; intraocular pressure (IOP) measurement; and fundus photography. Participants with hypertension, hypercholesterolemia, cerebrovascular disease, or cardiovascular disease were excluded to avoid confounding influences of these systemic conditions on ocular vascular health and inflammation. Notably, hypercholesterolemia can independently affect the vasculature and was therefore an exclusion criterion applied to both diabetic and control groups (19). Furthermore, additional exclusion criteria included refractive errors exceeding ±3.0 diopters, the presence of diabetic macular edema (DME) or any other fundus abnormality related to DR (since such findings would indicate diabetic retinopathy), a history of previous intraocular surgery or laser photocoagulation, treatment with anti-vascular endothelial growth factor (VEGF) agents, or any evidence of other chorioretinal diseases in the studied eye. Thus, any participant with DME or DR-related fundus changes was excluded to ensure that the diabetic group had no clinical signs of DR. A total of 61 patients (119 eyes) with T2DM, classified as having no DR (NDR) (i.e., diabetic patients without clinically detectable signs of DR), were recruited. In addition, 44 healthy individuals (82 eyes) with no history of diabetes or ocular disease were recruited as controls.

### Hematologic laboratory indices

Blood samples were collected from eligible participants for complete blood count (CBC) and biochemical examinations. The NLR, PLR, MLR, SII, and SIRI were calculated using blood routine indicators according to the following equations: NLR = neutrophil count/lymphocyte count; PLR = platelet count/lymphocyte count; MLR = monocyte count/lymphocyte count; SII = (neutrophil count × platelet count)/lymphocyte count; and SIRI = (neutrophil count × monocyte count)/lymphocyte count.

### Optical coherence tomography and optical coherence tomography angiography

SS-OCT and SS-OCTA images were obtained using a commercially available device (VG200, SVision Imaging Ltd., Luoyang, China), which operates at a central wavelength of 1,050 nm and a scanning speed of 200,000 A-scans per second. All scans were performed by the same experienced examiner (LS) and were subsequently reviewed for accuracy by two researchers (QX and QW). This wide-field system is capable of acquiring OCT images with a width of 26 mm, a depth of 12 mm, and a scanning angle of 130 degrees. The system offers an axial optical resolution of 3.8 μm and an axial digital resolution of 2 μm.

**Figure 1:**
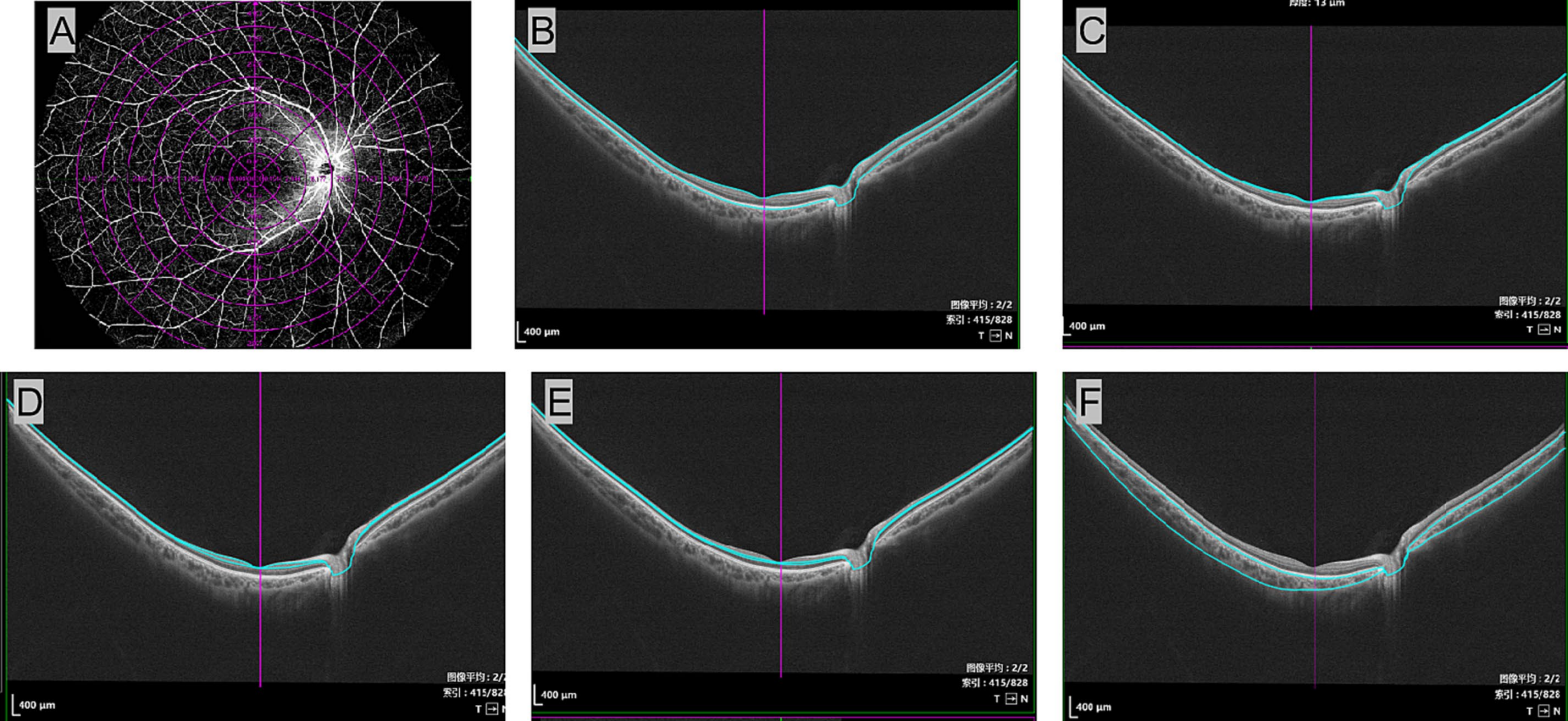
Automated method was used to segment retinal boundaries in each of the averaged B-scans in the Wide-field OCTA examination. A 26*21 mm OCTA pattern scan of the right eye of a patient, using the central macular recess as the center of the scan. The images were segmented into concentric circles with diameters of 3 mm, 6 mm, 9 mm, 12 mm, 15 mm, and 18 mm, based on the ETDRS grid **(A)**. The individual retinal layers were identified as follows: retina **(B)**, retinal nerve fiber layer **(C)**, ganglion cell layer + inner plexiform layer **(D)**, inner nuclear layer (INL) **(E)**, and choroid **(F)**. OCTA, optical coherence tomographic angiography; ETDRS, Early Treatment Diabetic Retinopathy Study.

Retinal and choroidal parameters were automatically obtained using the device’s built-in software. Images were segmented into concentric circles with diameters of 3 mm, 6 mm, 9 mm, 12 mm, 15 mm, and 18 mm, based on the Early Treatment Diabetic Retinopathy Study (ETDRS) grid. Retinal thickness was defined as the average distance from the internal limiting membrane to the outer border of Bruch’s membrane, while choroidal thickness was measured from the outer margin of the retinal pigment epithelium (RPE)–Bruch’s complex to the inner border of the choroidoscleral junction. The individual inner retinal layers were identified as follows, from inner to outer surface: retinal nerve fiber layer (RNFL), ganglion cell layer with inner plexiform layer (GCL+IPL), and inner nuclear layer (INL) (as shown in Figure 1).

**Figure 2:**
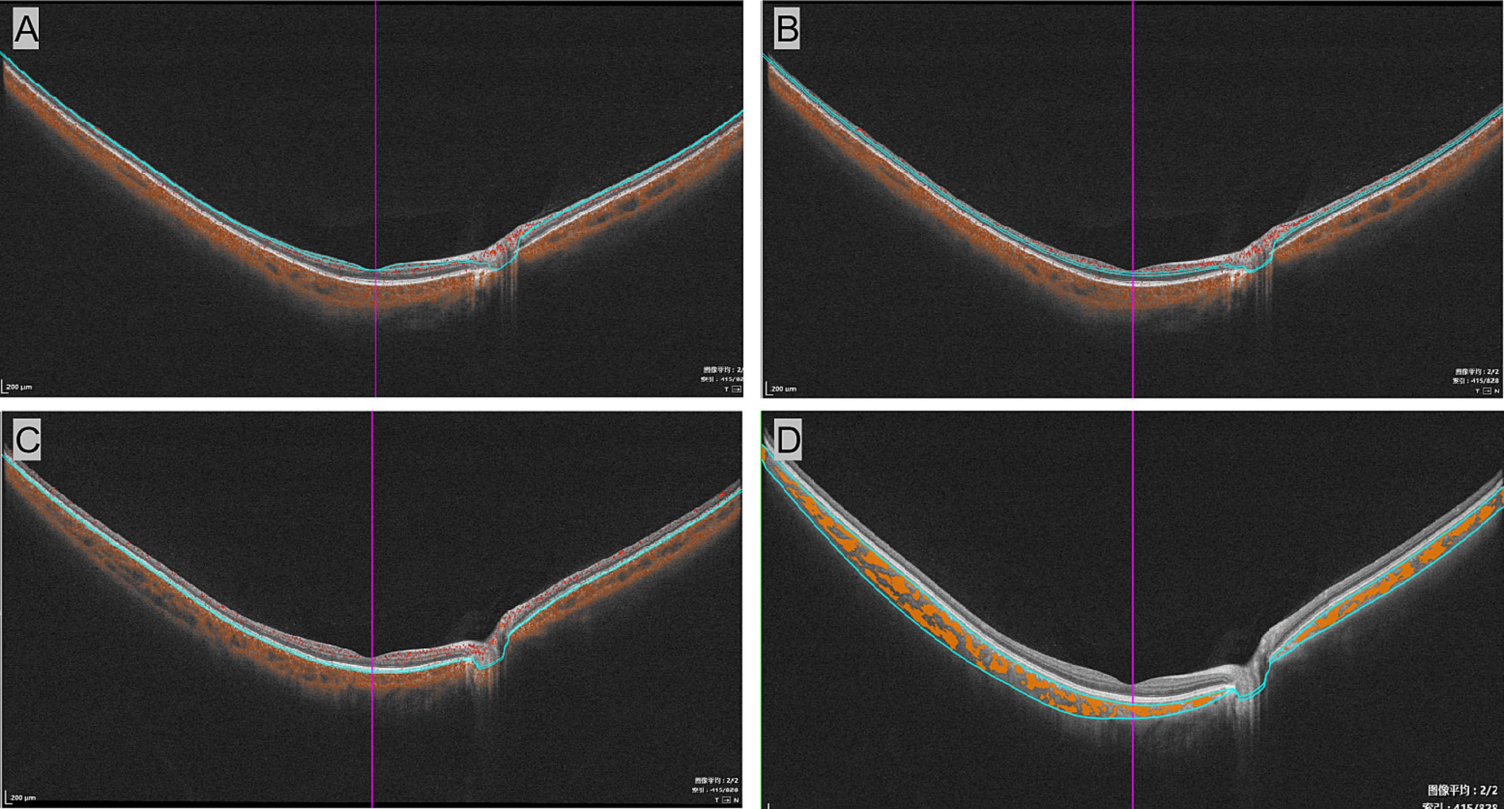
Segmentation of vascular plexuses in the wide-field optical coherence tomographic angiography examination. The B-mode images of the superficial vascular complex **(A)**, deep vascular complex **(B)**, and choriocapillaris **(C)**. **(D)** Displays measurements of the choroidal vascular index, with yellow marks representing the choroidal lumen.

The macula was segmented into three vascular plexuses—superficial vascular plexus (SVP) and deep capillary plexus (DCP)—along choriocapillaris (CC) using a built-in software algorithm. Vessel density (VD) refers to the percentage of the area occupied by blood vessels in a two-dimensional retinal projection image. Additionally, the choroidal vascular index (CVI) represents the ratio of the luminal area to the total choroidal area (as shown in Figure 2).

### Statistical analysis

All statistical analyses were performed using SPSS software (version 21.0, SPSS, Inc., Chicago, IL, USA). Continuous variables were expressed as means ± standard deviations (SD). To compare two groups, an independent samples t-test was used. The Shapiro–Wilk test was used to assess data normality. When the data did not meet the normality assumption, the Mann–Whitney U-test was applied instead of the t-test. The relationships between variables were assessed using either Pearson’s or Spearman’s correlation coefficients, depending on the data distribution. A *p*-value of less than 0.05 was considered statistically significant.

## Result

A total of 201 eyes were included in the study, with 61 patients (119 eyes) in the NDR group and 44 individuals (82 eyes) in the control group. The demographic and clinical characteristics of the participants are summarized in Table 1. The mean age was 65.90 ± 8.19 years, with 69.52% of the participants being women. There were no significant differences in age (*p* > 0.05) or gender distribution (*p* > 0.05) across the groups. Regarding immune-inflammatory markers, the NDR group showed significantly higher NLR (1.93 ± 0.85 vs. 1.60 ± 0.68, *p* < 0.01), SII (469.97 ± 357.61 vs. 378.82 ± 165.94, *p* < 0.05), and SIRI (0.62 ± 0.38 vs. 0.47 ± 0.26, *p* < 0.01) compared to the control group. Neutrophil count was also significantly higher in the NDR group (4.0 ± 1.38 vs. 3.04 ± 0.76, *p* < 0.01). However, there were no significant differences in PLR, MLR, lymphocyte count, monocyte count, or platelet count between the groups (*p* > 0.05).

**Table 1:** Comparison of the basic characteristics of the control group and patients with type 2 diabetes mellitus without clinical retinopathy.

Characteristics	All participants *n* = 105	T2DM without DR *n* = 61	Control group *n* = 44	*p*-values^a^
Age, years	65.90 ± 8.19	65.80 ± 7.60	66.02 ± 9.13	0.896
Gender, *n*%				0.696
Male	32 (30.48%)	20 (32.79%)	12 (27.27%)	
Female	73 (69.52%)	41 (67.21%)	32 (72.73%)	
Duration of T2DM, months	N/A	8.12 ± 5.38	N/A	N/A
HbA1c, %	N/A	6.55 ± 0.77	N/A	N/A
NLR	1.79 ± 0.8	1.93 ± 0.85	1.6 ± 0.68	0.005*
PLR	118.55 ± 43.18	114.73 ± 46.7	124.09 ± 37.07	0.131
MLR	0.15 ± 0.05	0.15 ± 0.05	0.15 ± 0.06	0.717
SII	432.79 ± 297.69	469.97 ± 357.61	378.82 ± 165.94	0.033*
SIRI	0.56 ± 0.34	0.62 ± 0.38	0.47 ± 0.26	0.003*
Neutrophils	3.61 ± 1.26	4.0 ± 1.38	3.04 ± 0.76	0*
Lymphocytes	2.18 ± 0.92	2.26 ± 1.07	2.06 ± 0.6	0.133
Monocytes	0.3 ± 0.09	0.31 ± 0.09	0.29 ± 0.07	0.058
Platelets	235.88 ± 52.63	233.51 ± 56.96	239.3 ± 45.74	0.445

Values are presented as the means ± standard deviations at baseline in different groups.

DR, diabetic retinopathy; HbA1c, hemoglobin A1C; MLR, monocyte-to-lymphocyte ratio; NA, not applicable; NLR, neutrophil-to-lymphocyte ratio; PLR, platelet-to-lymphocyte ratio; SII, systemic immune-inflammation index; SIRI, systemic inflammation response index; T2DM, type 2 diabetes mellitus.

a Statistical differences were analyzed between the patients with T2DM without DR and the normal control.

*p < 0.05 indicates a statistically significant difference.

**Figure 3:**
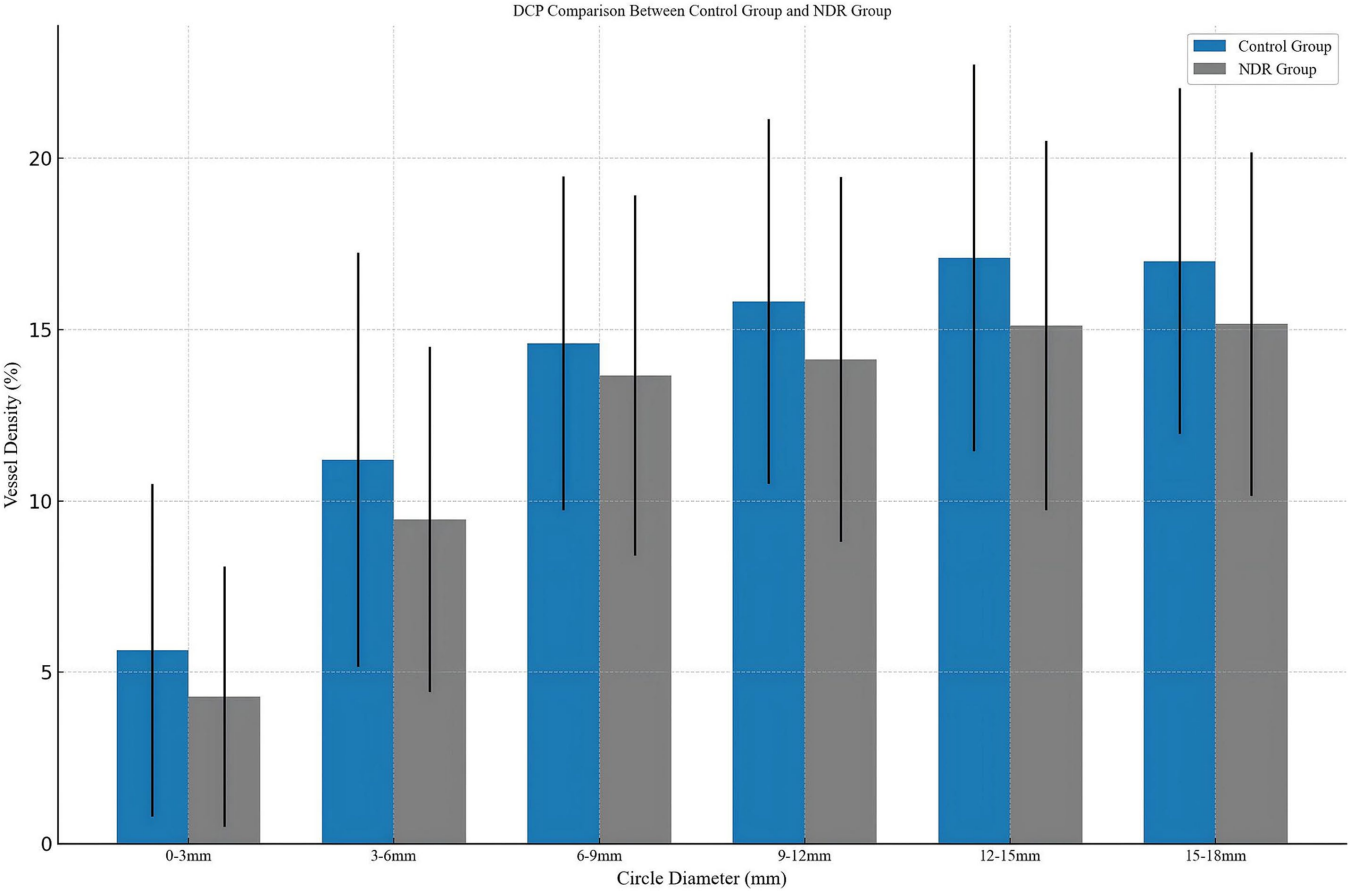
Comparison of patients with diabetes mellitus without clinical signs of DR and the control group. DCP, deep capillary plexus; NDR, type 2 diabetes mellitus patients without clinical diabetic retinopathy.

As shown in Table 2 and Figure 3, significant differences were found in the DCP, with the NDR group showing reduced perfusion in the 0–3 mm (4.29 ± 3.80 vs. 5.64 ± 4.85, *p* < 0.05), 3–6 mm (9.46 ± 5.04 vs. 11.20 ± 6.05, *p* < 0.05), 9–12 mm (14.13 ± 5.33 vs. 15.82 ± 5.33, *p* < 0.05), 12–15 mm (15.12 ± 5.39 vs. 17.10 ± 5.64, *p* < 0.05), and 15–18 mm (15.16 ± 5.02 vs. 17.00 ± 5.06, *p* < 0.05) regions (see Figure 3). No significant alterations were observed in SVP vessel density or choroidal parameters in patients with T2DM without clinical retinopathy across all regions (*p* > 0.05).

**Table 2:** Comparison of the optical coherence tomography angiography parameters between the control group and patients with type 2 diabetes mellitus without clinical retinopathy.

Parameters		Control group	T2DM without DR	*p*-values^a^
SVP	0–3 mm	60.85 ± 7.68	60.29 ± 10.12	0.651
	3–6 mm	60.82 ± 5.14	60.46 ± 6.31	0.662
	6–9 mm	36.46 ± 4.40	36.17 ± 4.04	0.641
	9–12 mm	26.15 ± 3.58	26.66 ± 4.20	0.360
	12–15 mm	24.89 ± 4.14	25.32 ± 4.99	0.506
	15–18 mm	22.28 ± 4.25	22.35 ± 4.79	0.920
DCP	0–3 mm	5.64 ± 4.85	4.29 ± 3.80	0.036*
	3–6 mm	11.20 ± 6.05	9.46 ± 5.04	0.033*
	6–9 mm	14.60 ± 4.87	13.66 ± 5.26	0.194
	9–12 mm	15.82 ± 5.33	14.13 ± 5.33	0.029*
	12–15 mm	17.10 ± 5.64	15.12 ± 5.39	0.014*
	15–18 mm	17.00 ± 5.05	15.16 ± 5.02	0.012*
CC	0–3 mm	5.38 ± 0.43	5.26 ± 0.57	0.107
	3–6 mm	16.80 ± 1.22	16.53 ± 1.28	0.139
	6–9 mm	28.04 ± 1.73	27.81 ± 1.93	0.378
	9–12 mm	39.48 ± 2.11	39.31 ± 2.42	0.599
	12–15 mm	51.95 ± 2.55	51.51 ± 3.03	0.269
	15–18 mm	62.49 ± 3.42	62.05 ± 3.77	0.381
CVI	0–3 mm	0.41 ± 0.07	0.40 ± 0.07	0.327
	3–6 mm	0.38 ± 0.06	0.36 ± 0.06	0.297
	6–9 mm	0.35 ± 0.06	0.34 ± 0.05	0.083
	9–12 mm	0.35 ± 0.06	0.34 ± 0.05	0.383
	12–15 mm	0.37 ± 0.06	0.36 ± 0.05	0.390
	15–18 mm	0.38 ± 0.05	0.37 ± 0.05	0.557

Values are presented as the means ± standard deviations at baseline in different groups.

CC, choriocapillaris; VI, choroidal vascular index; DCP, deep capillary plexus; DR, diabetic retinopathy; SVP, superficial vascular plexus; T2DM, type 2 diabetes mellitus.

a Statistical differences were analyzed between the patients with T2DM without DR and the normal control.

*p < 0.05 indicates statistically significant difference.

Furthermore, comparisons of OCT parameters between the control and NDR groups revealed significant differences, particularly in the central retina. The NDR group exhibited thicker retinal layers in the 0–3 mm region, including retinal thickness (315.31 ± 19.24 vs. 309.27 ± 17.04, *p* < 0.05), RNFL (26.60 ± 5.70 vs. 24.85 ± 2.36, *p* < 0.01), GCL+IPL (76.81 ± 7.37 vs. 73.47 ± 9.05, *p* < 0.01), and INL (42.35 ± 3.80 vs. 41.45 ± 2.17, *p* < 0.05). In contrast, no significant differences were observed in more peripheral regions (3–6 mm to 15–18 mm), suggesting that the effects of diabetes may be primarily localized to the central retina (Table 3).

**Table 3:** Comparison of the optical coherence tomography parameters between the control group and patients with type 2 diabetes mellitus without clinical retinopathy.

Parameters		Control group	T2DM without DR	*p*-values^a^
Retina	0–3 mm	309.27 ± 17.04	315.31 ± 19.24	0.021*
	3–6 mm	280.87 ± 14.94	283.43 ± 16.74	0.259
	6–9 mm	256.68 ± 14.56	257.45 ± 15.44	0.723
	9–12 mm	239.08 ± 13.23	239.98 ± 15.76	0.664
	12–15 mm	206.41 ± 7.29	206.79 ± 11.87	0.777
	15–18 mm	193.69 ± 7.68	195.18 ± 11.85	0.285
RNFL	0–3 mm	24.85 ± 2.36	26.60 ± 5.70	0.003*
	3–6 mm	42.88 ± 4.92	45.25 ± 7.86	0.009*
	6–9 mm	72.07 ± 11.08	74.87 ± 11.02	0.079
	9–12 mm	71.92 ± 11.42	73.48 ± 11.83	0.351
	12–15 mm	36.90 ± 5.35	38.15 ± 6.79	0.147
	15–18 mm	28.28 ± 3.91	30.09 ± 6.10	0.011*
GCL+IPL	0–3 mm	73.47 ± 9.05	76.81 ± 7.37	0.006*
	3–6 mm	57.54 ± 6.31	58.86 ± 6.06	0.140
	6–9 mm	32.64 ± 2.99	32.73 ± 3.61	0.844
	9–12 mm	25.11 ± 1.97	25.34 ± 2.76	0.498
	12–15 mm	24.69 ± 1.99	24.73 ± 2.84	0.917
	15–18 mm	23.98 ± 2.48	24.1 ± 3.78	0.780
INL	0–3 mm	41.45 ± 2.17	42.35 ± 3.80	0.035*
	3–6 mm	37.11 ± 1.64	37.48 ± 2.33	0.193
	6–9 mm	29.74 ± 1.05	29.85 ± 1.4	0.535
	9–12 mm	27.24 ± 1.07	27.33 ± 1.24	0.601
	12–15 mm	28.06 ± 1.41	27.93 ± 1.55	0.555
	15–18 mm	28.04 ± 1.55	28.03 ± 1.57	0.966
Choroid	0–3 mm	268.18 ± 95.62	275.28 ± 91.69	0.600
	3–6 mm	248.68 ± 78.50	254.28 ± 81.83	0.626
	6–9 mm	223.68 ± 59.41	227.94 ± 65.84	0.634
	9–12 mm	211.02 ± 49.20	215.85 ± 53.35	0.509
	12–15 mm	212.90 ± 43.11	216.19 ± 47.11	0.609
	15–18 mm	209.56 ± 36.54	212.11 ± 39.92	0.640

Values are presented as the means ± standard deviations at baseline in different groups.

DR, diabetic retinopathy; GCL, ganglion cell layer; INL, inner nuclear layer; IPL, inner plexiform layer; RNFL, retinal nerve fiber layer; T2DM, type 2 diabetes mellitus.

a Statistical differences were analyzed between the patients with T2DM without DR and the normal control.

*p < 0.05 indicates a statistically significant difference.

Table 4 presents the correlation analysis between inflammation markers (NLR, PLR, MLR, SII, and SIRI) and OCTA parameters in the NDR group across various retinal regions. SVP exhibited significant negative correlations with PLR and SIRI in the 3–6 mm and 6–9 mm regions (*p* < 0.05). Notably, NLR showed significant negative correlations with DCP in multiple regions, including the 3–6 mm (*r* = −0.21, *p* < 0.05), 6–9 mm (*r* = −0.18, *p* < 0.05), 9–12 mm (*r* = −0.32, *p* < 0.01), 12–15 mm (*r* = −0.31, *p* < 0.01), and 15–18 mm (*r* = −0.22, *p* < 0.05) regions. In the 9–12 mm and 12–15 mm regions, DCP exhibited negative correlations with MLR, SII, and SIRI (*p* < 0.05). In the choroid, NLR, MLR, and SIRI all showed significant negative correlations with CVI in several retinal regions, including the 0–3 mm (NLR: *r* = −0.23; MLR: *r* = −0.22; SIRI: *r* = −0.19), 3–6 mm (NLR: *r* = −0.27; MLR: *r* = −0.23; SIRI: *r* = −0.24), 6–9 mm (NLR: *r* = −0.28; MLR: *r* = −0.24; SIRI: *r* = −0.24), 9–12 mm (NLR: *r* = −0.24; MLR: *r* = −0.2), 12–15 mm (NLR: *r* = −0.26; MLR: *r* = −0.21; SIRI: *r* = −0.22), and 15–18 mm (NLR: *r* = −0.23; SIRI: *r* = −0.22) (*p* < 0.05). However, the absolute values of these correlation coefficients were generally low (approximately 0.2–0.3), indicating that the strength of these associations was weak despite reaching statistical significance.

**Table 4:** Correlations of inflammation markers and retinal optical coherence tomography angiography parameters in type 2 diabetes mellitus patients without clinical retinopathy.

Parameters		NLR		PLR		MLR		SII		SIRI	
		*r*-values	*p*-values	*r*-values	*p*-values	*r*-values	*p*-values	*r*-values	*p*-values	*r*-values	*p*-values
SVP	0–3 mm	−0.09	0.315	0.03	0.750	−0.09	0.353	−0.08	0.377	−0.21	0.021*
	3–6 mm	−0.21	0.027*	−0.02	0.867	−0.21	0.021*	−0.14	0.132	−0.3	0.001*
	6–9 mm	−0.21	0.021*	0.01	0.916	−0.11	0.222	−0.12	0.219	−0.21	0.027*
	9–12 mm	0.1	0.292	0.03	0.774	0.11	0.229	0.09	0.325	0.15	0.118
	12–15 mm	0.15	0.107	−0.01	0.913	0.18	0.045*	0.05	0.567	0.16	0.090
	15–18 mm	0.13	0.174	−0.06	0.498	0.2	0.028*	0.01	0.873	0.17	0.073
DCP	0–3 mm	−0.16	0.079	−0.05	0.612	−0.17	0.060	−0.09	0.352	−0.15	0.104
	3–6 mm	−0.21	0.023*	0.01	0.894	−0.22	0.016*	−0.09	0.321	−0.21	0.021*
	6–9 mm	−0.18	0.047*	0.05	0.591	−0.16	0.088	−0.06	0.536	−0.17	0.069
	9–12 mm	−0.32	0.001*	−0.11	0.247	−0.27	0.003*	−0.2	0.033*	−0.28	0.003*
	12–15 mm	−0.31	0.001*	−0.14	0.125	−0.25	0.006*	−0.21	0.022*	−0.25	0.006*
	15–18 mm	−0.22	0.020*	−0.13	0.171	−0.16	0.075	−0.16	0.089	−0.15	0.107
CC perfusion area	0–3 mm	0.08	0.405	0.07	0.432	0.03	0.724	0.03	0.748	−0.04	0.671
	3–6 mm	0.11	0.248	0.04	0.651	0.04	0.684	0.04	0.638	0.01	0.910
	6–9 mm	0.08	0.375	−0.02	0.797	0.02	0.831	0	0.982	0.01	0.942
	9–12 mm	0.11	0.244	−0.01	0.899	0.03	0.725	0.04	0.692	0.07	0.465
	12–15 mm	0.1	0.301	0	0.998	0.01	0.936	0.02	0.801	0.04	0.638
	15–18 mm	0.08	0.398	0.04	0.703	−0.01	0.921	0.02	0.809	0	0.958
CVI	0–3 mm	−0.23	0.014*	−0.05	0.612	−0.22	0.019*	−0.13	0.155	−0.19	0.040*
	3–6 mm	−0.27	0.003*	−0.02	0.861	−0.23	0.011*	−0.14	0.144	−0.24	0.008*
	6–9 mm	−0.28	0.002*	−0.03	0.731	−0.24	0.009*	−0.15	0.108	−0.24	0.008*
	9–12 mm	−0.24	0.010*	−0.08	0.380	−0.2	0.032*	−0.14	0.121	−0.18	0.059
	12–15 mm	−0.26	0.004*	−0.09	0.333	−0.21	0.020*	−0.18	0.049*	−0.22	0.016*
	15–18 mm	−0.23	0.014*	−0.08	0.408	−0.14	0.119	−0.19	0.043*	−0.22	0.017*

CC, choriocapillaris; CVI, choroidal vascular index; DCP, deep capillary plexus; MLR, monocyte-to-lymphocyte ratio; NA, not applicable; NLR, neutrophil-to-lymphocyte ratio; PLR, platelet-to-lymphocyte ratio; SII, systemic immune-inflammation index; SIRI, systemic inflammation response index; SVP, superficial vascular plexus.

*p < 0.05 indicates statistically significant difference.

As shown in Table 5, significant negative correlations were observed between the NLR and thickness in the inner retinal layers, particularly the GCL+IPL in the 3–6 mm region (*r* = −0.23, *p* < 0.05), 6–9 mm region (*r* = −0.25, *p* < 0.05), and the INL layer in the 3–6 mm (*r* = −0.20, *p* < 0.05) and 6–9 mm (*r* = −0.18, *p* < 0.05) regions. No significant correlations were found between PLR, MLR, and SII with OCT parameters. However, positive results in the analysis of SIRI and retinal layer thickness were observed, although these findings appeared to be incidental.

**Table 5:** Correlations of inflammation markers and retinal optical coherence tomography parameters in type 2 diabetes mellitus patients without clinical retinopathy.

Parameters		NLR		PLR		MLR		SII		SIRI	
		*r*-values	*p*-values	*r*-values	*p*-values	*r*-values	*p*-values	*r*-values	*p*-values	*r*-values	*p*-values
Retina	0–3 mm	−0.12	0.207	−0.04	0.657	−0.07	0.474	−0.07	0.473	−0.09	0.318
	3–6 mm	−0.19	0.044*	−0.1	0.301	−0.13	0.153	−0.11	0.242	−0.14	0.131
	6–9 mm	−0.04	0.634	−0.08	0.396	−0.04	0.675	−0.02	0.860	0.01	0.921
	9–12 mm	−0.1	0.286	−0.08	0.402	−0.02	0.841	−0.1	0.291	−0.09	0.319
	12–15 mm	−0.11	0.258	−0.13	0.152	−0.02	0.839	−0.12	0.199	−0.07	0.424
	15–18 mm	−0.11	0.249	−0.19	0.044	−0.03	0.748	−0.14	0.136	−0.05	0.562
RNFL	0–3 mm	0.02	0.839	−0.06	0.512	0.09	0.349	0	0.969	0.08	0.380
	3–6 mm	0.09	0.335	−0.07	0.443	0.05	0.624	0.04	0.636	0.15	0.112
	6–9 mm	0.15	0.106	0	0.990	0.06	0.537	0.12	0.203	0.18	0.050
	9–12 mm	−0.07	0.459	−0.04	0.674	−0.02	0.871	−0.1	0.307	−0.11	0.237
	12–15 mm	0.02	0.799	0.02	0.835	0.06	0.544	0.01	0.881	0.02	0.857
	15–18 mm	0.05	0.617	0.05	0.582	0.06	0.489	0.05	0.589	0.06	0.547
GCL+IPL	0–3 mm	−0.15	0.108	−0.05	0.595	−0.12	0.206	−0.09	0.329	−0.16	0.091
	3–6 mm	−0.23	0.012*	−0.03	0.773	−0.12	0.199	−0.11	0.262	−0.19	0.041*
	6–9 mm	−0.25	0.007*	−0.11	0.229	−0.1	0.277	−0.15	0.118	−0.15	0.101
	9–12 mm	−0.11	0.229	−0.11	0.230	−0.01	0.939	−0.06	0.557	0.02	0.869
	12–15 mm	−0.11	0.240	−0.12	0.189	0.01	0.905	−0.1	0.269	−0.03	0.789
	15–18 mm	−0.1	0.262	−0.16	0.077	−0.01	0.937	−0.12	0.206	−0.02	0.866
INL	0–3 mm	−0.09	0.316	−0.09	0.339	−0.06	0.548	−0.08	0.382	−0.08	0.415
	3–6 mm	−0.2	0.032*	−0.04	0.660	−0.12	0.213	−0.11	0.225	−0.15	0.097
	6–9 mm	−0.19	0.042*	−0.07	0.446	−0.07	0.432	−0.12	0.193	−0.12	0.192
	9–12 mm	0.11	0.222	0.1	0.282	0.17	0.063	0.15	0.098	0.19	0.047*
	12–15 mm	0.04	0.653	−0.01	0.910	0.12	0.190	0.02	0.811	0.08	0.387
	15–18 mm	0.09	0.322	0	0.980	0.1	0.276	0.07	0.452	0.11	0.258
Choroid	0–3 mm	−0.12	0.210	0.08	0.414	−0.1	0.263	−0.01	0.947	−0.12	0.202
	3–6 mm	−0.1	0.287	0.11	0.260	−0.09	0.341	0.01	0.890	−0.11	0.261
	6–9 mm	−0.06	0.492	0.17	0.074	−0.05	0.582	0.06	0.543	−0.07	0.437
	9–12 mm	−0.04	0.684	0.22	0.019*	−0.02	0.857	0.09	0.344	−0.05	0.617
	12–15 mm	−0.08	0.410	0.19	0.045*	−0.06	0.521	0.04	0.702	−0.1	0.292
	15–18 mm	−0.11	0.260	0.14	0.148	−0.09	0.349	−0.02	0.844	−0.14	0.139

GCL, ganglion cell layer; INL, inner nuclear layer; IPL, inner plexiform layer; MLR, monocyte-to-lymphocyte ratio; NA, not applicable; NLR, neutrophil-to-lymphocyte ratio; PLR, platelet-to-lymphocyte ratio; RNFL, retinal nerve fiber layer; SII, systemic immune-inflammation index; SIRI, systemic inflammation response index.

*p < 0.05 indicates statistically significant difference.

## Discussion

This research explores the relationship between systemic inflammation—reflected by elevated inflammatory markers such as NLR, MLR, SII, and SIRI—and neurovascular impairment, assessed through OCT and OCTA parameters, in the preclinical stages of DR. Specifically, individuals in the NDR group (diabetic patients without clinical DR) in our study exhibited a subtle increase in retinal thickness in the central retina, while showing significantly reduced vessel density in the DCP across all regions compared to the control group. Furthermore, significant associations were observed between these inflammatory markers and retinal vascular density, as well as choroidal structural changes, suggesting the role of inflammation in the initial pathophysiology of DR. Inflammation may therefore serve as both an early diagnostic biomarker and a potential therapeutic target in diabetes before retinopathy becomes clinically apparent.

While inflammation is critical, DR is a multifactorial and complex disease (20). Despite significant advances, the underlying mechanisms of this disease remain incompletely understood. Growing evidence suggests that a chronic systemic inflammatory state contributes to insulin resistance and the long-term complications of diabetes, playing a critical role in diabetic-associated retinal changes (7, 10). In line with previous studies, our research found significant differences in NLR, SII, and SIRI between the NDR group and the control group (15, 21, 22), supporting the notion that even before any signs of DR are visible, diabetic patients have elevated systemic inflammation.

These markers are commonly used to assess the risk and prognosis of various chronic inflammatory diseases (23), cardiovascular conditions (24), and cancers (25). Our findings showed that, even in the absence of clinical retinopathy, patients with T2DM may experience alterations in immune and inflammatory responses, potentially reflecting early disease processes. Notably, we observed no significant difference in PLR between the diabetic and control groups, indicating that PLR may be less sensitive than other composite indices in detecting early-stage DR-related inflammation. Similarly, Grossmann et al. reported elevated granulocyte and monocyte levels in blood samples from patients with T2DM, while lymphocyte levels remained relatively stable. Additionally, platelets were inversely associated with prediabetes, but not with diabetes (26). These findings align with our observation that certain components of the immune cell profile (neutrophils, represented in NLR, and platelets in SII/SIRI) differ between diabetics and healthy individuals even at the preclinical stage, whereas others (lymphocytes, monocytes, PLR) do not significantly differ.

To the best of our knowledge, this study is the first to investigate the relationship between inflammation markers and quantitative microvascular features of the eye using OCTA in diabetic patients without DR. This method enables a detailed assessment of diabetic-related changes in the ocular microcirculation over a large retinal area. Our findings suggest that significant alterations in the deep retinal vasculature may occur before overt DR, with more pronounced differences observed in peripheral retinal regions (9–12 mm to 15–18 mm). This emphasizes the necessity of wide-field OCTA imaging for diabetic patients, as it may provide more comprehensive insights into early vascular alterations that might be missed with conventional macular OCTA scans. Subgroup correlation analysis of this study revealed that blood inflammatory indexes, particularly the NLR, are associated with reduced vessel density in the SVP of the middle region, as well as in the DCP, excluding the central region. However, these correlations were relatively weak (*r* approximately −0.2 to −0.3), even though statistically significant, suggesting only a modest effect of systemic inflammation on local microvasculature. This may be due to the higher capillary density in the deep retinal vascular network, while the SVP (which is directly fed by retinal arterioles) may be better autoregulated and thus more resistant to mild systemic hypoxic or inflammatory insult (27).

While the relationship between NLR and DR has been extensively studied, our findings provide further insight, suggesting that systemic inflammation may contribute more to early microvascular changes than to neurodegenerative changes in the diabetic retina. NLR reflects the dynamic interaction between innate and adaptive immune responses in disease states, making it a reliable marker of systemic inflammation (28). Previous research indicates that NLR is a more stable inflammatory marker than any single leukocyte count (e.g., neutrophil, lymphocyte, or total white blood cell count) (29). Meta-analysis has suggested that NLR may primarily be involved in the early stages of DR, although it is not associated with DR grading (21).

In patients with T2DM, leukocytes are recruited to the vascular environment under conditions of oxidative stress and inflammation, leading to an increase in neutrophil counts and their adhesion to endothelial cells. This leads to vascular endothelial damage and widespread chronic inflammation (30). Elevated NLR has been proven to be associated with both microvascular and macrovascular complications of diabetes, as well as an increase in carotid intima-media thickness in T2DM patients (31). In the eye, our findings suggested that an elevated NLR correlates minimally with early neuronal damage but is more closely related to microvascular impairment, particularly affecting the deep retinal vasculature. These results highlight the potential of inflammation markers as early indicators of retinal microvascular changes in diabetes.

Additionally, more diffuse negative correlations were observed between inflammation markers and CVI. The choroid is the vascular layer supplying up to 85% of the ocular blood flow, with the foveola, in particular, relying entirely on it (32). Even in the absence of DR, ischemic changes in the choroidal vasculature may represent a key event in diabetic mellitus (33). However, traditional choroidal thickness is an unstable parameter influenced by various physiological, systemic, and local factors (34). In contrast, CVI is considered a more reliable marker for assessing choroidal vascular distribution and monitoring the progression of DR (35). Enface SS-OCT demonstrated vessel loss in Sattler’s layer in 30% of diabetic choroids and focal vessel narrowing in Haller’s layer in 51% of diabetic eyes, leading to a reduction in the proportion of the choroidal vasculature, known as CVI (36, 37). Our wide-field OCTA results are in agreement with these findings, showing a reduction in CVI in diabetics without DR, even though average choroidal thickness remained unchanged. This implies that early diabetic choroidopathy is characterized by microvascular rarefaction rather than gross thinning of the choroid, and that these changes are not confined to the subfoveal region but extend to the peripheral choroid.

The choroid, a site of significant immune activity, plays a pivotal role in the pathogenesis of various inflammatory disorders of the posterior segment (38). Inflammation appears to be a key factor in the progression of diabetic choroidopathy. The accumulation of leukocytes, along with an increase in inflammatory cells such as polymorphonuclear neutrophils, leads to vascular occlusion events and endothelial damage in the choroid (39). Using wide-field OCTA, we confirmed that systemic inflammation is associated with reduced choroidal vascularity (lower CVI) in diabetic patients without DR, despite there being no significant change in choroidal thickness. This indicates that inflammatory damage in diabetes may first manifest as a loss of choroidal vascular caliber or density rather than atrophy of the choroid, highlighting the insidious impact of inflammation on the choroidal circulation even before retinopathy becomes clinically apparent.

Several limitations of the current study should be acknowledged. First, the retrospective cross-sectional design of the study is a major limitation, as it precludes establishing causal relationships and does not allow assessment of disease progression over time. Second, the relatively small sample size may limit the generalizability of the findings. Third, we did not perform multivariable analyses to adjust for potential confounders (such as age, HbA1c level, or duration of diabetes), which might have influenced the observed associations. Furthermore, OCTA imaging is susceptible to artifacts, including automated segmentation errors and projection artifacts, which could affect the accuracy of the results. Taken together, these limitations suggest that our findings should be interpreted with caution. Future studies should address these limitations by using a larger cohort, adopting a longitudinal (prospective) design with follow-up to track progression, and controlling for additional confounders through multivariate analysis.

## Conclusion

In conclusion, we used wide-field SS-OCTA to investigate retinochoroidal neuron structural and microcirculation characteristics in T2DM patients without DR. Compared to healthy controls, eyes with preclinical DR (NDR) exhibited a significant reduction in deep retinal vascular density, as well as subtle changes in retinal thickness and choroidal vascularity, despite a normal ophthalmoscopic examination. Inflammatory indices, particularly NLR, MLR, and SIRI, were closely associated with retinal NVU impairment and choroidal vascular damage in T2DM patients. These markers may serve as valuable tools for the early detection and monitoring of diabetic retinopathy and choroidopathy, and might also represent potential targets for early therapeutic intervention in order to prevent or delay the onset of DR. Further research is needed to explore the underlying mechanisms of inflammation-induced ocular microvascular changes, which could provide novel insights for the early diagnosis and treatment of diabetic retinal diseases.

## Data Availability

The raw data supporting the conclusions of this article will be made available by the authors, without undue reservation.

## Glossary

CBC: Complete blood count
CC: Choriocapillaris
CVI: Choroidal vascularity index
DCP: Deep capillary plexus
DR: Diabetic retinopathy
ETDRS: Early Treatment Diabetic Retinopathy Study
GCL: Ganglion cell layer
INL: Inner nuclear layer
IPL: Inner plexiform layer
MLR: Monocyte-to-lymphocyte ratio
NLR: Neutrophil-to-lymphocyte ratio
NVU: Neurovascular unit
OCTA: Optical coherence tomography angiography
PLR: Platelet-to-lymphocyte ratio
RPE: Retinal pigment epithelium
RNFL: Retinal nerve fiber layer
SD: Standard deviations
SII: Systemic immune-inflammation index
SIRI: Systemic inflammation response index
SS-OCTA: Swept-source optical coherence tomography angiography
SVP: Superficial vascular plexus
T2DM: Type 2 diabetes mellitus
VD: Vessel density
